# Targeted Inactivation of *p12^Cdk2ap1^*, CDK2 Associating Protein 1, Leads to Early Embryonic Lethality

**DOI:** 10.1371/journal.pone.0004518

**Published:** 2009-02-20

**Authors:** Yong Kim, Jim McBride, Lauren Kimlin, Eung-Kwon Pae, Amit Deshpande, David T. Wong

**Affiliations:** 1 Division of Oral Biology and Medicine, Dental Research Institute, School of Dentistry, University of California Los Angeles, Los Angeles, California, United States of America; 2 UCLA's Jonsson Comprehensive Cancer Center, University of California Los Angeles, Los Angeles, California, United States of America; 3 Molecular Biology Institute, University of California Los Angeles, Los Angeles, California, United States of America; 4 Division of Head and Neck Surgery/Otolaryngology, University of California Los Angeles, Los Angeles, California, United States of America; 5 Henry Samueli School of Engineering, University of California Los Angeles, Los Angeles, California, United States of America; 6 Section of Orthodontics, School of Dentistry, University of California Los Angeles, Los Angeles, California, United States of America; Health Canada, Canada

## Abstract

Targeted disruption of murine *Cdk2ap1*, an inhibitor of CDK2 function and hence G1/S transition, results in the embryonic lethality with a high penetration rate. Detailed timed pregnancy analysis of embryos showed that the lethality occurred between embryonic day 3.5 pc and 5.5 pc, a period of implantation and early development of implanted embryos. Two homozygous knockout mice that survived to term showed identical craniofacial defect, including a short snout and a round forehead. Examination of craniofacial morphology by measuring Snout Length (SL) vs. Face Width (FW) showed that the *Cdk2ap1^+/−^* mice were born with a reduced SL/FW ratio compared to the *Cdk2ap1^+/+^* and the reduction was more pronounced in *Cdk2ap1^−/−^* mice. A transgenic rescue of the lethality was attempted by crossing *Cdk2ap1^+/−^* animals with *K14-Cdk2ap1* transgenic mice. Resulting *Cdk2ap1^+/−:K14-Cdk2ap1^* transgenic mice showed an improved incidence of full term animals (16.7% from 0.5%) on a *Cdk2ap1^−/−^* background. Transgenic expression of *Cdk2ap1* in *Cdk2ap1^−/−:K14-Cdk2ap1^* animals restored SL/FW ratio to the level of *Cdk2ap1^+/−:K14-Cdk2ap1^* mice, but not to that of the *Cdk2ap1^+/+:K14-Cdk2ap1^* mice. Teratoma formation analysis using mESCs showed an abrogated *in vivo* pluripotency of *Cdk2ap1^−/−^* mESCs towards a restricted mesoderm lineage specification. This study demonstrates that *Cdk2ap1* plays an essential role in the early stage of embryogenesis and has a potential role during craniofacial morphogenesis.

## Introduction

*CDK2AP1* was initially identified as a cancer-related gene by using hamster oral cancer model [1]. *CDK2AP1* is a highly conserved and ubiquitously expressed gene located on human chromosome 12q24 and is a 115-aa nuclear polypeptide that is downregulated in ∼70% of oral cancers [2], [3]. Murine *Cdk2ap1* with only three amino acid deviations from the human *CDK2AP1* is located at chromosome 5 [4], [5]. We have obtained significant amount of data demonstrating potential cellular function of CDK2AP1 *in vitro* and *in vivo* [5], [6], [7], [8], [9], [10], [11]. In addition to its role as a cell cycle regulatory molecule through two important cellular partners: CDK2 and DNA polymerase-alpha/primase, we have shown that CDK2AP1 has a role in TGF-β induced growth arrest, cisplatin induced genotoxicity, and cellular apoptosis [6], [7], [8], [10], [11]. Recently, we have shown the *in vivo* tumor regression effect of CDK2AP1 with reducing proliferation and increasing apoptotic indices in a xenograft mouse model of head and neck cancer [10]. Furthermore, we have demonstrated that overexpression of *Cdk2ap1* in a transgenic mouse model resulted in gonadal atrophy, seminiferous tubule degeneration, and folliculogenesis abnormalities *in vivo* [12]. Despite the exact *in vivo* physiological role of Cdk2ap1 remains to be determined, there are several indirect evidences that implicate its role in development. *Cdk2ap1* has been identified as one of stem cell specific genes that are enriched in both embryonic and adult stem cells [13]. More interestingly, it has been reported through microarray analysis that *Cdk2ap1* has been categorized as one of genes that are expressed in an early stage preimplantation embryos and its expression gradually decreases as the embryo further develops [14]. Through a global expression map of cell cycle regulators in mitosis and meiosis, *Cdk2ap1* was found as one of genes with postnatal testis maturation-associated decrease [15]. In addition, *Cdk2ap1* mRNA has been found to be elevated upon estrogen treatment during early implantation process, suggesting its role in uterine decidualization where the cells stop proliferation and start differentiation [16].

Proper development of organisms including vertebrates requires spatial and temporal orchestration between many different molecules. Uncontrolled or deleted expression of certain gene can cause abnormal embryo development leading to lethality [17], [18], [19], [20], [21]. These are classes of molecules that are known to play essential roles during development. However though their associated molecular mechanisms have been extensively studied, our understanding towards developmental process especially during the early embryogenesis remains largely elusive. Early stage of the embryo development is defined as a period of rapid cellular proliferation to suffice the needed number of cells in a short period of time. This is most likely related to either silenced or reduced activity of cell cycle regulatory pathways observed in embryonic stem cells [22], [23], [24], [25]. Several well-known cell cycle regulatory molecules including pRb are known to remain inactive during early gestation period until embryonic stem cells start to differentiate into specified cell lineages [26], [27], [28]. The physiological function of a given molecule or its significance in development *in vivo* can be studied by taking a knockout approach in mouse. In this paper, we examined the developmental role of Cdk2ap1 through a knockout approach via a specific deletion of *Cdk2ap1* in mouse to understand physiological significance of Cdk2ap1 during development. Our data clearly demonstrate that *Cdk2ap1* is an essential gene during early embryo development since the targeted homozygous disruption of *Cdk2ap1* resulted in the lethality of the early embryos. Interestingly, significant craniofacial abnormality was observed in the *Cdk2ap1* knockout model, implicating its potential role of Cdk2ap1 in skeletal morphogenesis.

## Materials and Methods

### Targeted disruption of p12^Cdk2ap1^ and generation of knockout mice

Murine embryonic stem cells (ES-LW1) were grown on a gelatin coated plate and maintained in DMEM supplemented with 15% FBS, 0.1 µM β-mercaptoethanol, 1% L-Glutamine, 0.2% (v/v) LIF, 1% (v/v) PSF, and 0.1% (v/v) gentamycin sulfate. Generation of *Cdk2ap1* knockout mESCs was described by Kim et al. [5]. Established two *Cdk2ap1^+/−^* cells were independently injected into blastocysts from C57/BL6. Chimeric animals were identified by genotyping and Southern analysis and populated to establish *Cdk2ap1^+/−^* mouse line. All the animals were maintained and taken care of under the husbandry guidelines supervised by the UCLA Chancellor's Animal Research Committee.

### Genotyping and Southern analysis of Cdk2ap1 knockout mice

Tail biopsies from weaned animals were collected according to the guidelines from UCLA Animal Research Committee. Genomic DNA was extracted by incubating in 17 mM Tris-HCl (pH 7.5), 17 mM EDTA, 170 mM NaCl, 0.85% SDS, and 0.2 mg/ml proteinase K overnight at 55°C. Final DNA pellet was dissolved in TE and subjected to a multiplex PCR analysis by using Advantage 2 PCR kit (Clontech, Palo Alto, CA). Primer set 1 [CDU2 (5′GCCTTCTTGACGAGTTCTTCTGAG3′) and CDD2 (5′TGGATGTGGAATGTGTGCGAG3′)] amplifies a 460 bp fragment within the cytosine deaminase gene in the targeting vector and primer set 2 [I2U (5′ATGGGGATGGATGTGTGAGAGG3′) and I2D (5′TGGGTTCAAGGAAGTGGACTAATG3′)] generates a 239 bp fragment from intron 2 which is absent in a successful homologous recombination). For Southern analysis, 5 µg of DNA was digested with Bsu36I resulting in 8.4 and 5.6 kb fragments.

### Timed pregnancy analysis and genotyping

For timed pregnancy analysis, heterozygous females were mated with heterozygous males with or without hormonal stimulation. At day 2.5 or 3.5 dpc, females were sacrificed and the embryos were flushed out of the uterus. Genomic DNA was isolated by using blastocyst lysis buffer (10 mM Tris-HCl, pH 8.5, 50 mM KCl, 2 mM MgCl_2_, 0.45% NP-40, 0.45% Tween 20, 60 µg/ml proteinase K) and subjected to multiplex PCR analysis with or without genomic DNA amplification by using GenomiPhi DNA Amplification kit (GE Healthcare, Piscataway, NJ) according to the manufacturer's instruction. For later gestation time points, the embryos were fixed in 4% paraformaldehyde, rinsed under tap water, stored in 70% EtOH and paraffin-embedded for sectioning onto metal LCM slides. LCM slides were deparaffinized 5 min in xylene and rehydrated in 100% EtOH for 2 min, 95% EtOH for 2 min, 70% EtOH for 2 min, 50% EtOH for 2 min and dH_2_O for 2 min. Slides were stained with methyl green for 30 sec and washed in dH_2_O for 1–5 min. Finally slides were air dried and a portion of embryo was microdissected into PCR caps containing 20 µl of digestion buffer (Arcturus DNA Extraction Kit). Tissue was digested overnight as suggested by Arcturus protocol. A 5 µl of digest was used per PCR reaction by using Advantage 2 PCR kit (Clontech, Palo Alto, CA). Two different sets of primers were used for genotyping analysis: For homologous recombinant DNA, CD3F (TGTCGTATCCCAACGGTGAAGC) and CD1R (CCAGGGCGAAGGTTTTATGC) or CD3F and CD3R (ACATCATCGTGACCAAAGCAGACG) were used. For the wild type DNA, Int4F (TTGCTTTGCTTGTTTCCTGGGC) and IntR (GGCTGGGGTTTGGCTCATAGAATC) were used.

### Generation of Cdk2ap1^−/−:K14-Cdk2ap1^ hybrid animals

Transgenic *K14-Cdk2ap1* mice were generated as described previously [12]. Heterozygous *Cdk2ap1^+/−^* mice were mated with *K14-Cdk2ap1* mice and pups were analyzed by PCR based genotyping on tail biopsies. Confirmed heterozygous *Cdk2ap1^+/−:K14-Cdk2ap1^* mice were intercrossed to examine the viability of homozygous *Cdk2ap1^−/−:K14-Cdk2ap1^*. In addition, the expression of transgenic *Cdk2ap1* was confirmed by RT-PCR analysis with total RNA from ear-snips. Briefly, frozen earsnips were powdered and mixed with lysis buffer. Total RNA was purified by using RNeasy mini kit from Qiagen (Valencia, CA). For RT-PCR analysis, equal amount of total RNA was subjected to reverse transcription by using Superscript III RT and oligo(dT) primer. The synthesized cDNA was amplified by using a pair of primers specific to mouse *Cdk2ap1*. In addition, GAPDH primer was used for normalization.

### Blastocyst outgrowth analysis

Embryos were harvested at 3.5 dpc as described for timed pregnancy analysis and cultured in gelatin coated 96 well plate in blastocyst culture medium ((DMEM, 20% FBS, 2 mM glutamine, 4 mg/ml BSA, 0.1 mM β-ME, PSF). Culture was maintained for 5 days and the hatching and outgrowth of blastocyst was photomicrographed for comparison. At the end of culture, cells were lysed in blastocyst lysis buffer (10 mM Tris-HCl, pH 8.5, 50 mM KCl, 2 mM MgCl_2_, 0.45% NP-40, 0.45% Tween 20, 60 µg/ml proteinase K) for isolation of total genomic DNA. Genotyping analysis was performed as described above.

### Mesenchymal cell culture

Primary bone marrow mesenchymal cell were isolated and established by flushing bone marrows from the tibiae, femora, and humeri of 4 week-old animals with cold DMEM using a 27-gauge needle. Cells were then washed with cold PBS and cultured to confluence in a 100 mm plate in α-MEM with 10% FBS. At confluence cells were collected for further analysis.

## Results

### Targeted disruption of p12^CDK2-AP1^ in mice leads to early embryonic lethality

**Figure 1:**
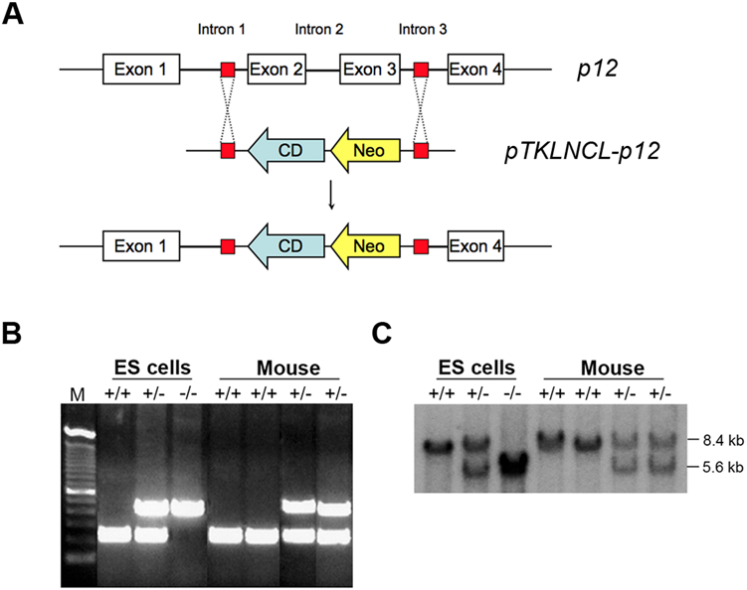
Generation of *Cdk2ap1* knockout mice. A. Targeting vector construct *pTKLNCL-Cdk2ap1* was designed to knockout *Cdk2ap1* exon2 and 3 by homologous recombination. Cytosine deaminase (CD) and neomycin resistance (*Neo*) genes were placed in to disrupt *Cdk2ap1* gene in mouse chromosome 5. B. Multiplex genotyping analysis was used to screen recombinant ES cells as described in Materials and Methods. The top band of 460 bp is amplified from cytosine deaminase in the targeting vector and the bottom band of 239 bp is amplified from the introns of wild type *Cdk2ap1*. Mouse tail DNA was also analyzed for screening of *Cdk2ap1* knockout mice by multiplex PCR. C. The multiplex PCR genotyping data was confirmed by Southern blot analysis. The wild type *Cdk2ap1* showed 8.4 kb fragment, while the recombinant showed 5.6 kb fragment.

**Figure 2:**
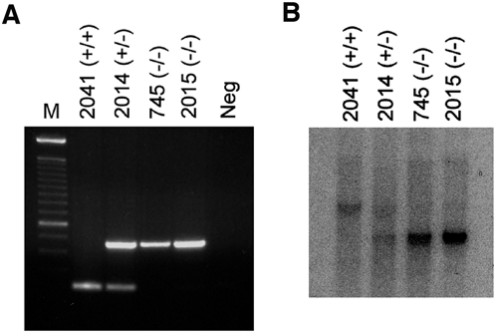
Genotyping of survived homozygous *Cdk2ap1* knockout mice. Two mice (M745 and F2015) were born live out of 399 pups analyzed from crossing of heterozygous *Cdk2ap1* knockout mice. A. The genotype of both animals was analyzed by multiplex PCR on tail DNA with no DNA negative control. B. The PCR genotyping result was also confirmed by Southern analysis. Both M745 and F2015 mice showed homozygous knockout genotype compared to WT type (F2041) and heterozygous (F2014) animals.

Significant amount of data has been gathered demonstrating the importance of Cdk2ap1 in cellular regulation *in vitro* and also *in vivo* [5], [8], [9], [10]. However, the normal physiological role of Cdk2ap1 has not been fully elucidated. As a way of evaluating the importance of Cdk2ap1 in the animal development and physiology, we generated a conventional knockout mouse model. Heterozygous and homozygous *Cdk2ap1* knockout murine embryonic stem cells were generated as reported previously [5]. Targeting vector was designed to recombine with the introns 1 and 4, resulting in the substitution of the exons 2 and 3 with *neo^r^* and cytosine deaminase genes (Fig. 1). Electroporated ES-LW1 cells (129sv background) were screening for gaining of resistance to G418. Selected heterozygous *Cdk2ap1* knockout cells were further screened for homozygous knockout through mitotic crossing over event under high dose of G418 selection. The expression of *Cdk2ap1* was examined by RT-PCR, northern and western analysis. Homozygous knockout cells showed a complete absence of *Cdk2ap1* expression [5]. To generate *Cdk2ap1* knockout mice, heterozygous *Cdk2ap1* knockout cell line 272 or 284 was independently injected into the blastocysts from C57/B6 mice. The injected blastocysts were transferred into the uterus of the pseudo-pregnant mouse. Chimeras were verified by PCR analysis on tail DNA and southern blot analysis. Chimeric animals were mated to generate heterozygous *Cdk2ap1* knockout mouse lines and heterozygosity was verified by multiplex PCR and southern analysis on tail biopsies (Fig. 1). We then attempted to generate homozygous line by mating proven heterozygous pairs. From extensive breeding and genotyping analysis, we have found the knockout of *Cdk2ap1* resulted in very high penetrating rate of embryonic lethality. As shown in Table 1, we had 2/399 (from 84 litters) homozygous knockout mice. The genotyping result of the two survived homozygous *Cdk2ap1* knockout mice is shown in Fig. 2. To define a window of lethality and gain insight into the role of Cdk2ap1 during embryogenesis, we have performed a detailed timed pregnancy analysis by using heterozygous animals. The result showed that there were 2.5 and 3.5 dpc embryos genotyped as homozygous knockout (7/24 and 11/66, respectively), but we have not observed any viable *Cdk2ap1^−/−^* embryos after 3.5 dpc. These data suggest that the lethality occurs after 3.5 dpc blastocyst stage and a knockout of *Cdk2ap1* may have an effect on the proper hatching of blastocyst, the implantation of embryo, or very early differentiation of embryos.

**Table 1:** Timed pregnancy analysis by Het x Het matings.

Age	Litter	Total Number	Genotype			Resorbed
			+/+	+/−	−/−	
4 weeks	56	399	137	260	2	N/A
E10.5	5	48	21	27	0	6
E7.5	5	29	12	17	0	4
E5.5	5	24	8	16	0	N/A
E3.5	9	66	21	34	11	N/A
E2.5	4	24	5	12	7	N/A

**Figure 3:**
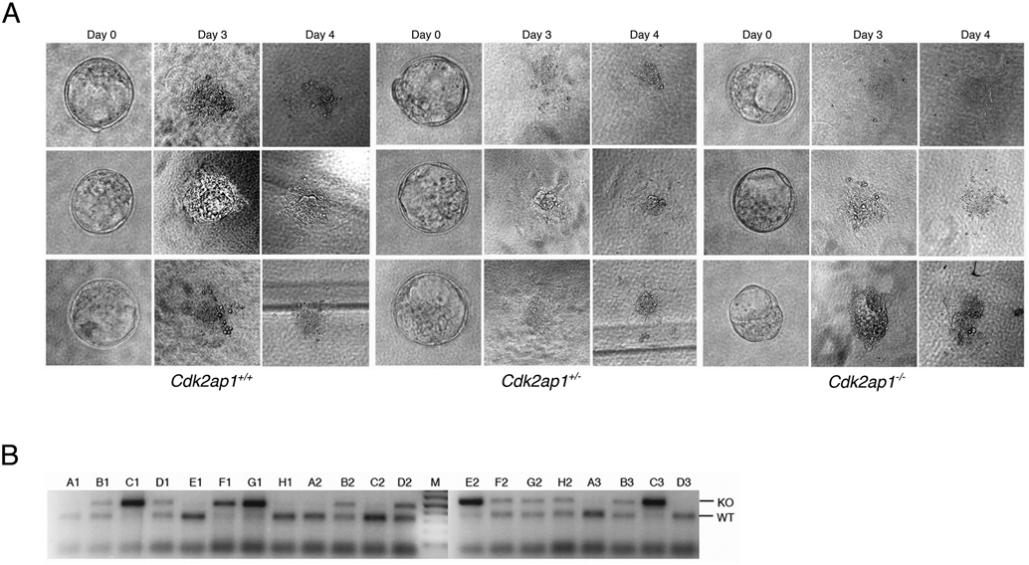
Blastocyst outgrowth analysis of *Cdk2ap1* knockout embryos. To examine the hatching and outgrowth of *Cdk2ap1^−/−^* embryos, blastocyst outgrowth analysis was performed. Heterozygous females and males were mated for timed pregnancy analysis and E3.5 blastocysts were collected. A. Individual blastocyst was cultured in a 96 well plate for 4 days. Photomicrograph was taken every day to monitor the hatching and outgrowth of blastocysts. B. After final recording of morphology, outgrown cells were lysed and genomic DNA was isolated for multiplex genotyping analysis as described in Materials and Methods. The PCR products were analyzed on a 1.5% agarose gel and the genotype was matched to the phenotype from A.

To determine if the deletion of *Cdk2ap1* has any effect on hatching process of blastocysts, we have performed blastocyst outgrowth analysis. Embryos at E3.5 were collected from timed pregnancy analysis and cultured in vitro to observe hatching and outgrowth of embryonic stem cells. At the end of observation, cells were genotyped to match with the phenotypes. As shown in Fig. 3, blastocysts properly hatched and outgrew regardless of the genotype. There were no significant changes observed among *Cdk2ap1^+/+^*, *Cdk2ap1^+/−^*, and *Cdk2ap1^−/−^* blastocysts. This result suggests that the lethality from the deletion of *Cdk2ap1* is not due to the abrogation in the hatching process.

### Rescue of lethality by crossing Cdk2ap1^+/−^ with transgenic K14-Cdk2ap1

**Figure 4:**
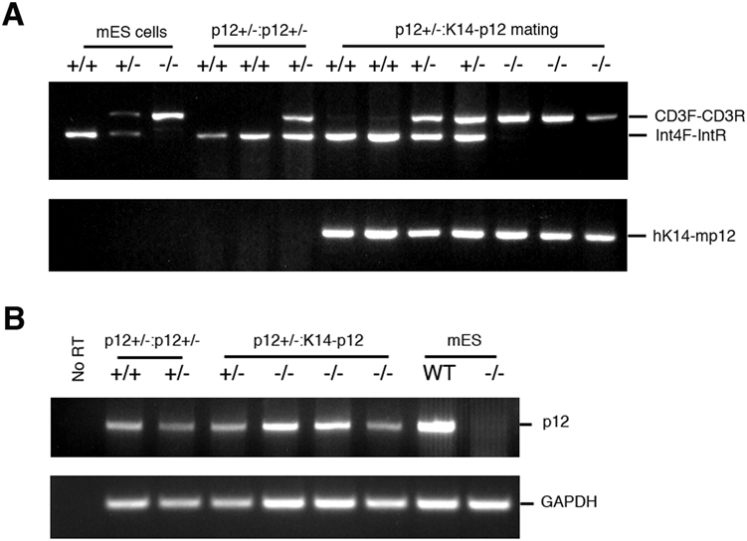
Genotyping and expression analysis of *Cdk2ap1^−/−:K14-Cdk2ap1^* hybrids. Hybrid mice were generated by crossing heterozygous *Cdk2ap1* knockout mice and *K14-Cdk2ap1* transgenic mice. A. The resulting pups were genotyped to identify hybrids by using PCR. Wild type *Cdk2ap1* gene was amplified by using Int4F and IntR primers against the *Cdk2ap1* intron sequence. The recombinant *Cdk2ap1* was detected by using CD3F and CD3R primers against cytosine deaminase gene. In addition, the presence of transgenic *Cdk2ap1* was detected by using *hK14* and *mCdk2ap1* primers against human K14 promoter and mouse *Cdk2ap1* transgene. By intercrossing heterozygous *Cdk2ap1^+/−^:K14-Cdk2ap1* mice, we were able to generate homozygous *Cdk2ap1^−/−^:K14-Cdk2ap1* mice. Mouse ESCs (*Cdk2ap1^+/+^*, *Cdk2ap1^+/−^*, and *Cdk2ap1^−/−^*) and pups (WT and heterozygous) from mating of heterozygous *Cdk2ap1* knockout mice were used as controls. B. The expression of *Cdk2ap1* was examined by RT-PCR analysis on total RNA from earsnips as described in Materials and Methods. Compared to no RT or *Cdk2ap1^−/−^* mESC control, comparable amount of *Cdk2ap1* mRNA was detected in *Cdk2ap1^−/−^:K14-Cdk2ap1* mouse tissues, which showed that the expression of *Cdk2ap1* was achieved from the *Cdk2ap1* transgene in the mice with *Cdk2ap1^−/−^* genotype.

The process of generating knockout mice involves an extensive *in vitro* screening of recombinant embryonic stem cells, including an extended period of culturing and a selection with high dose of antibiotics. This could potentially impose the surmount level of pressure to the cells and lead to an unwanted bias in the analysis. The most appropriate approach to avoid this kind of bias would be using a conditional knockout model. This is currently in progress and we have already generated heterozygous floxed *Cdk2ap1* knockout mice by crossing chimeras. Meanwhile, to ensure that the lethality we observed with homozygous *Cdk2ap1* knockout mice was due to the specific inactivation of the gene, we tried to rescue the lethal phenotype of *Cdk2ap1^−/−^* mice by taking a transgenic approach. To complement the loss of *Cdk2ap1* expression in *Cdk2ap1* knockout mice, we crossed the heterozygous *Cdk2ap1* knockout (*Cdk2ap1^+/−^*) mice with the *K14-Cdk2ap1* transgenic (*Cdk2ap1^+/−:K14-Cdk2ap1^*) mice that overexpress *Cdk2ap1* by using human *keratin 14* promoter [12]. We first generated and identified F1 hybrid mice with *Cdk2ap1^+/−^* genotype and *K14-Cdk2ap1* transgene integrated into the genome (*Cdk2ap1^+/−:K14-Cdk2ap1^*). As shown in Fig. 4A, we were able to generate hybrid mice of *Cdk2ap1^−/−^* genotype with *K14-Cdk2ap1* transgene integrated into the genome. The resulting heterozygous *Cdk2ap1^+/−:K14-Cdk2ap1^* hybrids were then intercrossed to generate homozygous *Cdk2ap1* knockout with *K14-Cdk2ap1* transgene (*Cdk2ap1^−/−:K14-Cdk2ap1^*). We investigated the effect of transgenic rescue on the development of *Cdk2ap1^−/−:K14-Cdk2ap1^* mice in terms of survival and proper development. The resulting weaned pups of 4 weeks of age were analyzed by genotyping on tail biopsies. We have analyzed 36 pups from 11 litters and the transgenic rescue approach resulted in an improved birth of homozygous *Cdk2ap1* knockout mice (Table 2). The rate of the birth of *Cdk2ap1^−/−^* was increased to 16.7% from 0.5%. The expected incidence of homozygous pups should be 25% based on Mendelian genetics. Therefore, it is about 67% of the expected number, which means while the rescue was not achieved with 100% efficiency. It still represents significantly improved rescue of lethality. During this hybrid mating process, we have not obtained any homozygous *Cdk2ap1* knockout mice without the integrated *Cdk2ap1* transgene. This appears especially important because we can rule out the strain dependent genetic modifier effect with this finding. To see if the integrated transgene through the hybrid approach is expressed in the mice, we examined *Cdk2ap*1 expression in *Cdk2ap1^−/−:K14-Cdk2ap1^* hybrid animals by RT-PCR analysis on total RNA isolated from ear-snips. As shown in Fig. 4B, we detected the expression of *Cdk2ap1* mRNA in *Cdk2ap1^+/+^* and *Cdk2ap1^+/−^* mice from mating of heterozygous *Cdk2ap1^+/−^* mice. The *Cdk2ap1^+/−:K14-Cdk2ap1^* mouse from mating of *Cdk2ap1^+/−:K14-Cdk2ap1^* hybrids also expressed *Cdk2ap1* mRNA. We observed that there was *Cdk2ap1* mRNA expressed in *Cdk2ap1^−/−:K14-Cdk2ap1^* hybrids, probably due to the expression of transgenic *Cdk2ap1*. As anticipated the *Cdk2ap1^−/−:K14-Cdk2ap1^* mice also expressed considerable level of *Cdk2ap1* mRNA detected by RT-PCR, while *Cdk2ap1^−/−^* mESCs used as a control did not show any expression of *Cdk2ap1* mRNA. These evidence confirm that the complementary expression of *Cdk2ap1* has been achieved in *Cdk2ap1^−/−:K14-Cdk2ap1^* hybrids.

**Table 2:** Transgenic rescue result from genotyping of *Cdk2ap1^+/−:K14-Cdk2ap1^* mating.

Age	Litter	Total Number	Genotype		
			+/+	+/−	−/−
4 weeks	11	36	10	20	6

### Craniofacial abnormalities in survived Cdk2ap1^−/−^ mice

**Figure 5:**
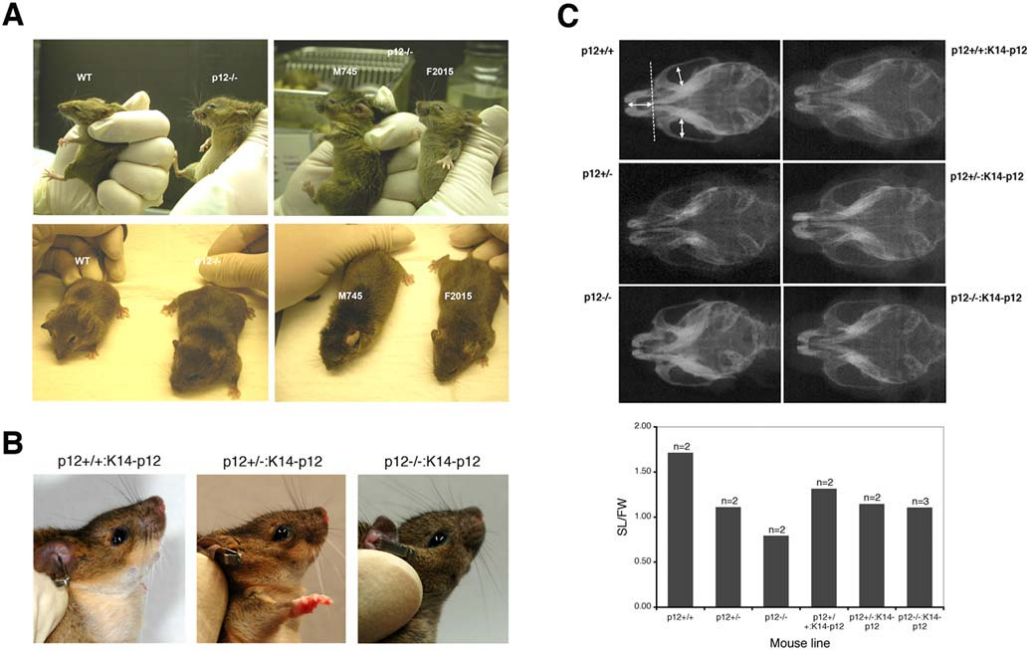
Craniofacial abnormality of survived *Cdk2ap1^−/−^* mice. A. The craniofacial morphology was noticeably different between wild type (WT) and *Cdk2ap1^−/−^* mice. Two survived *Cdk2ap1^−/−^* mice (M745 and F2015) were also compared side-by-side and both mice showed similar craniofacial abnormality compared to WT. B. The complement expression of *Cdk2ap1* in *Cdk2ap1^−/−^* mice by transgenic approach resulted in an increased incidence of homozygous *Cdk2ap1* knockout mice. The rescued mice (*Cdk2ap1^−/−:K14-Cdk2ap1^*) also showed the recovery of the craniofacial deformity to the state of the heterozygous (*Cdk2ap1^+/−:K14-Cdk2ap1^*) mice. C. The craniofacial differences between animals were analyzed by measuring the Snout Length (SL) and Facial Width (FW) ratio measured on the X-ray films obtained in a standardized head position as indicated by the arrows. The average ratio was depicted and compared between *Cdk2ap1^+/+^* and *Cdk2ap1^−/−^* mice, and also the hybrid animals with *Cdk2ap1^+/+:K14-Cdk2ap1^*, *Cdk2ap1^+/−:K14-Cdk2ap1^*, and *Cdk2ap1^−/−:K14-Cdk2ap1^* genotype.

One of the interesting findings was that the two homozygous *Cdk2ap1* knockout mice (M745 and F2015) born and lived exhibited similar craniofacial defects with a noticeable short snout and a round forehead compared to wild type (Fig. 5A). We further reasoned that if Cdk2ap1 plays a specific role in craniofacial development, the hybrid mice with transgenic *Cdk2ap1* expression should overcome the craniofacial abnormality observed in *Cdk2ap1^−/−^* mice. As shown in Fig. 5B, the hybrid *Cdk2ap1^−/−:K14-Cdk2ap1^* mice did not show much phenotypic difference from *Cdk2ap1^+/+:K14-Cdk2ap1^* or *Cdk2ap1^+/−:K14-Cdk2ap1^* mice. To examine this phenotype in more detail, we have performed X-ray cephalometry of the animals. We analyzed the morphometric difference of the facial skeletons of the animals by comparing the Snout Length vs. Face Width (SL/FW) (Fig. 5C). As shown in the X-ray result, *Cdk2ap1^−/−^* animal showed a noticeable reduction of SL/FW ratio compared to the wild type (Table 3). The wild type *Cdk2ap1^+/+^* mice showed an average SL/FW ratio of 1.72, while *Cdk2ap1^−/−^* mice showed an average SL/FW ratio of 0.80, which is only 47.0% of the wild type. This clearly shows that the survived *Cdk2ap1^−/−^* mice were born with a reduced snout length compared to wild type mice. Furthermore, we also observed the reduction of SL/FW ratio in heterozygous *Cdk2ap1* knockout mice compared to WT. The SL/FW ratio in heterozygous *Cdk2ap1* knockout mice was 1.11, which is 65.0% of the wild type. To see if the abnormal craniofacial morphology is specifically due to the loss of Cdk2ap1 expression, we also examined the SL/FW ratio of *Cdk2ap1^−/−:K14-Cdk2ap1^* mice rescued by the transgenic complementation of *Cdk2ap1* expression with *K14-Cdk2ap1* mice. As summarized in Table 3, we observed that the average SL/FW ratio of *Cdk2ap1^−/−^* hybrid mice (SL/FW = 1.10) were returned to that of *Cdk2ap1^+/−^* hybrid mice (SL/FW = 1.18) after complementation of *Cdk2ap1* expression by transgenic *Cdk2ap1*. In both *Cdk2ap1^+/−:K14-Cdk2ap1^* and *Cdk2ap1^−/−:K14-Cdk2ap1^* mice, the SL/FW ratio was still smaller than *Cdk2ap1^+/+:K14-Cdk2ap1^* mice (89.0% and 83.0% of the *Cdk2ap1^+/+:K14-Cdk2ap1^* mice, respectively). Comparison of the SL/FW ratios from heterozygous mice and homozygous mice without or with transgenic complementation showed that the ratio was improved in the hybrid animals (increased from 73% without complementation to 93% with complementation), which implies that the complementary expression of *Cdk2ap1* in *Cdk2ap1^−/−:K14-Cdk2ap1^* mice rescued the craniofacial deformity to the state of the heterozygous *Cdk2ap1^+/−:K14-Cdk2ap1^* mice.

**Table 3:** Snout Length (SL) and Facial Width (FW) ratio.

Mouse line	*Cdk2ap1^+/+^*	*Cdk2ap1^+/−^*	*Cdk2ap1^−/−^*	*Cdk2ap1^+/+^:K14-Cdk2ap1*	*Cdk2ap1^+/−^:K14-Cdk2ap1*	*Cdk2ap1^−/−^:K14-Cdk2ap1*
SL/FW	1.82	1.07	0.67	1.42	1.18	1.22
	1.61	1.15	0.92	1.21	1.18	0.98
						1.12
Average	1.72	1.11	0.80	1.32	1.18	1.10
Vs. +/+	1	0.65	0.47	1	0.89	0.83

### Expression of Cdk2ap1 in mesenchymal stem cells in rescued hybrid animals

**Figure 6:**
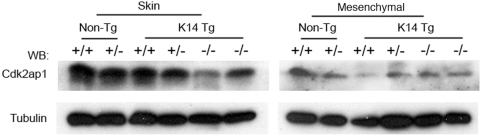
Expression of *K14-Cdk2ap1* in mesenchymal cells in the rescued mice. A. Expression of *K14-Cdk2ap1* transgene was detected in skin tissues from *Cdk2ap1^−/−^:K14-Cdk2ap1* mice. Earsnips from non-transgenic or transgenic mice (*Cdk2ap1^+/+^:K14-Cdk2ap1*, *Cdk2ap1^+/−^:K14-Cdk2ap1*, *Cdk2ap1^−/−^:K14-Cdk2ap1*) were harvested and subjected to Western analysis with anti-Cdk2ap1 antibody. The loading was confirmed by staining with anti-tubulin antibody. B. To examine if the *K14-Cdk2ap1* transgene is expressed in mesenchymal lineage cells, bone marrow was isolated from *Cdk2ap1^−/−^:K14-Cdk2ap1* mice. Cultured mesenchymal stem and progenitor cells were subjected to Western analysis to examine the expression of *K14-Cdk2ap1* in *Cdk2ap1^−/−^:K14-Cdk2ap1* mice.

Since K14 promoter is known to be active in epithelial lineage cells, it is necessary to demonstrate if the rescue of craniofacial defect by transgene is through its expression in mesenchymal cells. To address this, we have isolated mesenchymal stem and progenitor cells from bone marrow in rescued hybrid animals. As shown in Fig. 6, the expression of *Cdk2ap1* was detected in skin tissues and mesenchymal cells from *Cdk2ap1^+/+^* and *Cdk2ap1^+/−^* mice. It was also found that the expression of transgenic *Cdk2ap1* was detected in both skin tissues and mesenchymal cells from *Cdk2ap1^−/−:K14-Cdk2ap1^* mice. This demonstrates that the expression of *K14-Cdk2ap1* transgene is not limited to epithelial cell lineages, but also it is actively expressed in mesenchymal cells from bone marrow of *Cdk2ap1^−/−:K14-Cdk2ap1^*.

### Altered in vivo pluripotency in Cdk2ap1^−/−^ mESCs

**Figure 7:**
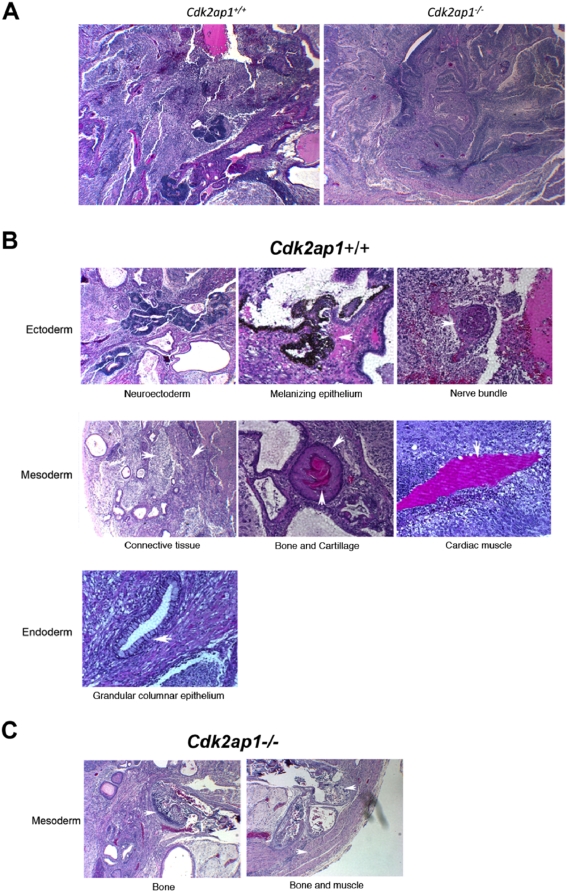
*Cdk2ap1^−/−^* mESCs showed an abrogated *in vivo* pluripotency. *In vivo* pluripotential competence of *Cdk2ap1^−/−^* mESCs was evaluated by teratoma formation analysis. *Cdk2ap1^+/+^* or *Cdk2ap1^−/−^* mESCs were transplanted into the testis of SCID mice in duplicate as described by Conway et al. (29). After 4 weeks, tumors were extracted and subjected to fixation and sectioning. The slides were stained with H&E and examined under bright field microscope. A. Gross examination of teratoma sections from *Cdk2ap1^+/+^* and *Cdk2ap1^−/−^* mESCs (×4 magnification). B. Specified three lineages committed from *Cdk2ap1^+/+^* mESCs. C. A restricted commitment of *Cdk2ap1^−/−^* mESCs to a certain mesoderm lineage.

Our extensive *in vitro* studies with *Cdk2ap1^−/−^* mESCs showed that Cdk2ap1 plays a role in mESC differentiation by modulating the expression of stem cell marker genes and also differentiation-related genes, especially involved in trophoblast and mesoderm differentiation (Kim et al., manuscript in review). The *in vivo* pluripotency of *Cdk2ap1^−/−^* mESCs was examined by teratoma formation analysis. Either *Cdk2ap1^+/+^* or *Cdk2ap1^−/−^* mESCs (0.5–1.0×10^6^ cells) were transplanted into the testis of SCID mice. After 4–6 weeks, mice were euthanized and tumors were extracted for histological analysis [29]. As shown in Fig. 7, both *Cdk2ap1^+/+^* and *Cdk2ap1^−/−^* mESCs formed teratomas. However, there was noticeable difference between tumors from *Cdk2ap1^+/+^* and *Cdk2ap1^−/−^* mESCs at low magnification. Tumors from *Cdk2ap1^−/−^* mESCs did not show definable differentiation compared to tumors from *Cdk2ap1^+/+^* mESCs (Fig. 7A). *Cdk2ap1^+/+^* mESCs showed proper differentiation and commitment into three different lineages as shown in the Fig. 7B. Interestingly, only types of tissues we observed in tumors from *Cdk2ap1^−/−^* mESCs were bones and muscles, which are mesoderm lineage tissues (Fig. 7C). This result demonstrates that the deletion of Cdk2ap1 led to a compromised *in vivo* pluripotency in mESCs. This is very intriguing finding that suggests a potential role of Cdk2ap1 in mouse embryo development and also it provides us an insight on a potential mechanism of the early embryonic lethality in *Cdk2ap1^−/−^* mice. Taken together, this suggests that the deletion of *Cdk2ap1* could lead to an abrogated differentiation of mESCs during early embryo development and further result in the early embryonic lethality potentially through defect in cellular differentiation and development.

## Discussion

Data presented in this paper clearly demonstrate that *Cdk2ap1* is an essential gene in the proper development of mouse embryos. It remains to be seen what kind of role Cdk2ap1 precisely play during embryogenesis. We have obtained significant amount of data supporting that the lethality is due to an abrogated differentiation of mESCs, which is involved in placenta development (Kim et al., manuscript in review). We also demonstrated that a specific deletion of *Cdk2ap1* leads to the aberrant craniofacial development. This finding may be noteworthy even though we only have two survived animals showing this phenotype due to the high penetrance of lethality in *Cdk2ap1^−/−^* animal. It is well known that the transforming growth factor-beta (TGF-β) and Smad pathway is involved in craniofacial morphogenesis [30], [31], [32], [33]. We have extensive lines of evidence demonstrating the function of Cdk2ap1 in cell cycle regulation, apoptosis, and also growth arrest and tumor regression [5], [8], [9], [10], [34]. Interestingly, we have also demonstrated that the functional involvement of Cdk2ap1 in TGF-β induced growth arrest. It has been shown that *Cdk2ap1* is activated by TGF-β-Smad2 and mediates TGF-β induced growth arrest in normal diploid cells and the loss of *Cdk2ap1* expression in squamous cell carcinoma is correlated with disrupted TGF-β-Smad signaling pathway [9], [11]. The role of the TGF-β family in normal embryonic development, specifically in development of the craniofacial region, has been well documented [30], [33]. The function of these molecules is vital to development of the secondary palate. They regulate maxillary and palate mesenchymal cell proliferation and extracellular matrix synthesis. The function of this growth factor family is particularly critical in that perturbation of either process results in a cleft of the palate. The cellular and phenotypic effects of TGF-β on embryonic craniofacial tissue have been extensively examined, but the specific genes that function as downstream mediators of TGF-β in maxillary/palatal development are poorly defined. We have used a transgenic approach to rescue the lethality by crossing with the *K14-Cdk2ap1* transgenic mice. It is well documented that *K14* promoter is active in epithelial tissues, including skin, palate, tongue, and ovary [35], [36], [37], [38], [39], [40]. In the previous paper demonstrating the phenotype of *K14-Cdk2ap1* mice, we have observed the overexpression of *Cdk2ap1* in epithelial tissues [12]. Now the question we have is how the overexpression of *Cdk2ap1* directed in epithelial tissues in *K14-Cdk2ap1* mice rescued craniofacial defects in mesenchymal origin. It is known that epithelial cells can undergo transition to mesenchymal cells through the process called epithelial-to-mesenchymal transition (EMT) [41], [42]. The roles of EMT have been described in embryonic development and morphogenesis. In embryonic development, it is known that EMT affects tissues on a global scale, such as gastrulation and also formation of a three-layered embryo. It has an effect on organogenesis of heart, musculoskeletal system, and craniofacial structures such as palate [42]. It is also known that TGF-β signaling is significantly associated with the induction and maintenance of EMT [43], [44], [45]. Based on these, we speculate that the overexpression of *Cdk2ap1* in epithelial cells in *Cdk2ap1* knockout and *K14-Cdk2ap1* hybrid embryo could have an effect on the embryo development and on the craniofacial development possibly through the EMT process.

From *in vitro* study using *Cdk2ap1* knockout mouse embryonic stem (mES) cells, we are beginning to unveil potential roles of Cdk2ap1 during differentiation process (Kim et al., manuscript in review). We have observed significantly compromised differentiation potential in *Cdk2ap1^−/−^* mESCs and we are in the process of detailing a mechanism that underlies the cellular and molecular significance of Cdk2ap1 during the early embryogenesis. Even though this is the first report demonstrating the importance of Cdk2ap1 in the embryogenesis, other lines of evidences also suggest the potential involvement of Cdk2ap1 in stem cell biology and also implantation process [13], [14], [16]. Once our ongoing attempt to generate an inducible conditional *Cdk2ap1* knockout mouse model is complete, we will be able to address standing questions regarding the essential role of Cdk2ap1 in the animal development and its underlying mechanisms.
